# Deletion of *taf1* and *taf5* in zebrafish capitulate cardiac and craniofacial abnormalities associated with TAFopathies through perturbations in metabolism

**DOI:** 10.1242/bio.059905

**Published:** 2023-07-13

**Authors:** Jamison Leid, Ryan Gray, Peter Rakita, Andrew L. Koenig, Rohan Tripathy, James A. J. Fitzpatrick, Charles Kaufman, Lilianna Solnica-Krezel, Kory J. Lavine

**Affiliations:** ^1^ Center for Cardiovascular Research, Division of Cardiology, Department of Medicine, Washington University School of Medicine, St. Louis, MO 63110, USA; ^2^ Departments of Nutritional Sciences, Dell Pediatrics Research Institute, University of Texas at Austin, Austin, TX 78723, USA; ^3^ Departments of Neuroscience and Cell Biology, Washington University Center for Cellular Imaging, Washington University School of Medicine, St. Louis, MO 63110, USA; ^4^ Department of Developmental Biology, Washington University School of Medicine, St. Louis, MO 63110, USA; ^5^ Division of Oncology, Department of Medicine, Washington University School of Medicine, St. Louis, MO 63110, USA; ^6^ Department of Immunology and Pathology, Washington University School of Medicine, St. Louis, MO 63110, USA

**Keywords:** TAFopathy, TAF1, TAF5, Heart development, Craniofacial development, Metabolism

## Abstract

Intellectual disability is a neurodevelopmental disorder that affects 2-3% of the general population. Syndromic forms of intellectual disability frequently have a genetic basis and are often accompanied by additional developmental anomalies. Pathogenic variants in components of TATA-binding protein associated factors (TAFs) have recently been identified in a subset of patients with intellectual disability, craniofacial hypoplasia, and congenital heart disease. This syndrome has been termed as a TAFopathy and includes mutations in TATA binding protein (TBP), *TAF1*, *TAF2*, and *TAF6*. The underlying mechanism by which TAFopathies give rise to neurodevelopmental, craniofacial, and cardiac abnormalities remains to be defined. Through a forward genetic screen in zebrafish, we have recovered a recessive mutant phenotype characterized by craniofacial hypoplasia, ventricular hypoplasia, heart failure at 96 h post-fertilization and lethality, and show it is caused by a nonsense mutation in *taf5*. CRISPR/CAS9 mediated gene editing revealed that these defects where phenocopied by mutations in *taf1* and *taf5*. Mechanistically, *taf5-/-* zebrafish displayed misregulation in metabolic gene expression and metabolism as evidenced by RNA sequencing, respiration assays, and metabolite studies. Collectively, these findings suggest that the TAF complex may contribute to neurologic, craniofacial, and cardiac development through regulation of metabolism.

## Teaser

**Summary:** Pathogenic variants in components of TATA-binding protein associated factors (TAFs) have been identified in patients with intellectual disability, craniofacial hypoplasia, and congenital heart disease. Our findings demonstrate that the TAF complex contributes to neurologic, craniofacial, and cardiac development through regulation of metabolism.

## INTRODUCTION

Birth defects are a major cause of morbidity and mortality worldwide. Between 3-5% of live born infants have birth defects including cardiac, craniofacial, and neurodevelopmental abnormalities that each contribute to adverse perinatal outcomes (Webber et al., 2015). While significant progress has been made identifying environmental contributors to birth defects, our understanding of their genetic basis remains incomplete (Kirby, 2017).

Transcriptional dysregulation represents an important mechanism leading to birth defects (Prescott and Wilkie, 2007). Gene regulatory networks that establish cell states, pattern tissues, and regulate cell differentiation and organ maturation are controlled by thousands of transcription factors, cofactors, and chromatin regulators. Failure to regulate these programs leads to a diverse array of developmental syndromes, including but not limited to, growth and intellectual disability, limb deformities, craniofacial anomalies, and congenital heart defects (Jenkins et al., 2007; de Soysa et al., 2019). It is increasingly recognized that pathogenic variants in key transcription factors are responsible for many developmental syndromes. These ‘general regulator’ transcription factors control gene expression and chromatin accessibility through selective recruitment of RNA Polymerase II and epigenetic machinery to specific genomic loci (Clark et al., 2006; Chen et al., 2022).

Recently, pathogenic variants in TATA Binding Protein Associated Factors (TAFs) key components of transcription factor TFIID have been identified in a subset of patients with intellectual disability, craniofacial hypoplasia, and congenital heart disease (O'Rawe et al., 2015). This syndrome has been termed as a ‘TAFopathy’ and includes mutations in TATA Binding Protein (*TBP*), *TAF1*, *TAF2*, *TAF6*, *TAF8*, and *TAF13* (Rooms et al., 2006; Tawamie et al., 2017; Hellman-Aharony et al., 2013; Alazami et al., 2015). The underlying mechanism by which TAFopathies give rise to neurodevelopmental, craniofacial, and cardiac abnormalities is not understood.

TFIID is a large complex comprised of multiple proteins including TBP and multiple TAFs. The complete TFIID complex and holo-TFIID (lacking TBP) bind to distinct DNA sequences (Wu and Chiang, 2001). Previous studies have identified specific DNA sequences to which TAFs bind within initiator and downstream promoter elements (Juven-Gershon et al., 2006; Juven-Gershon and Kadonaga, 2010). TAFs also interact with enhancers through bromodomain motifs (Lehmann et al., 2012). TAF1 and TAF5 are core components of TFIID complexes. TAF1 binds to DNA and TBP, forming a complex that recruits RNA Polymerase II at sites of transcription. TAF5 acts as a scaffold protein important for the assembly of TFIID components (Bieniossek et al., 2013; Wang et al., 2014). Further adding to the complexity, distinct TFIID complexes consisting of unique TAF combinations may mediate the effect of different transcriptional activators (Brou et al., 1993; Asturias, 2009; Louder et al., 2016).

Here, we report a novel zebrafish model of TAFopathies caused by nonsense mutations in *taf5*. *taf5^−/−^* zebrafish display reduced survival, heart failure, and facial and cardiac hypoplasia and fail to thrive. These findings were recapitulated in *taf1^−/−^* zebrafish. Mechanistically, we reveal roles for TAF1 and TAF5 in coordinating metabolic programs essential for craniofacial and cardiac development.

## RESULTS

### *cora^stl325/stl325^* embryos show signs of early-onset heart failure

**Fig. 1.**
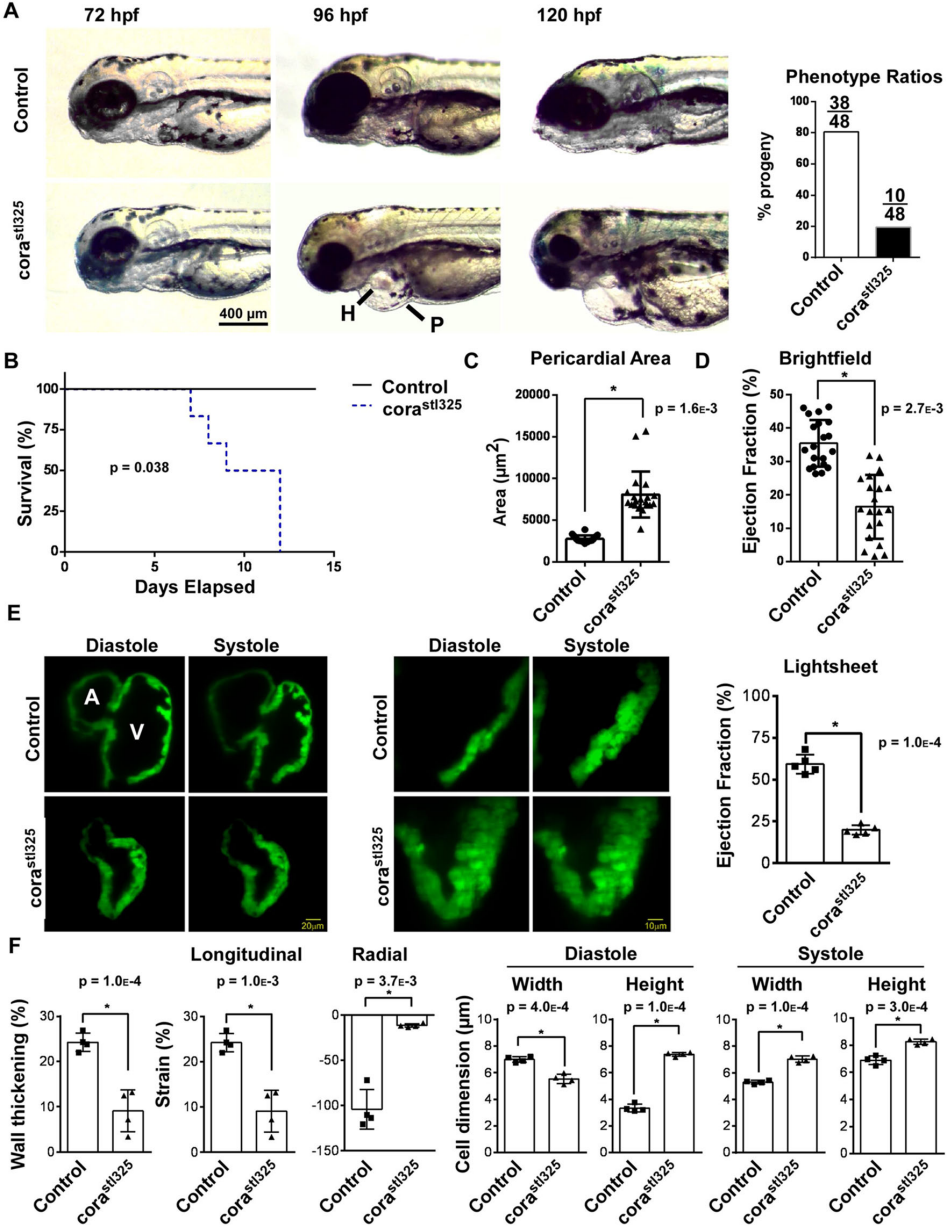
***corast^l325/stl325^* embryos display reduced survival and heart failure**. (A) Light microscopy showing the emergence of pericardial edema between 72 and 120 h post fertilization (hpf). P, pericardium; H, heart. Phenotype ratios are presented on the right. (number of trials=4; number of samples per trial=48) (B) Survival curve of *cora^stl325^* embryos relative to their unaffected clutchmates (number of trials=4; number of samples per trial=48). (C) Quantification of light microscopy images measuring area of the pericardium (number of trials=4; number of samples per trial=48). (D) Quantification of light microscopy images measuring ejection fraction (number of trials=4; number of samples per trial=48). (E) Lightsheet microscopy generated images of 96 hpf embryos harboring the cmlc::GFP transgenic reporter. Hearts are shown in systole and diastole. A, atrium; V, ventricle. Quantification of ejection fraction (*n*=4). (F) Quantification of changes in cell dimension using lightsheet-generated videos (*n*=4).

We recovered a mutation from an ENU mutagenesis screen that displayed a small heart and craniofacial hypoplasia and lethality, which we referred to as *corazoncito^stl325^ (cora^stl325^)* (Gray et al., 2021). Affected embryos demonstrate evidence of heart failure in Mendelian ratios suggestive of a recessive allele (Fig. 1A). To characterize the cardiac phenotype in detail, we first observed the temporal progression of heart failure in progeny obtained from a *cora^stl325/+^* carrier self-cross. *cora^stl325/stl325^* embryos displayed a collapsed heart and pericardial edema beginning at 96 h post-fertilization (hpf). *cora^stl325/stl325^* hearts were visually indistinguishable from unaffected clutch mates at 72 hpf (Fig. 1A). We observed 100% mortality of embryos that displayed the *corazoncito* phenotype within 2 weeks post-fertilization (Fig. 1B). Quantitatively, *cora^stl325/stl325^* embryos had a nearly three-fold increase in pericardial surface area compared to unaffected clutch mates (Fig. 1C). Measurement of ejection fraction by bright field imaging indicated that *corazoncito* embryos displayed evidence of systolic ventricular dysfunction (Fig. 1D).

To further characterize defects in cardiac function, we crossed *cora^stl325/+^* to the cardiomyocyte-specific fluorescent reporter, *Tg(cmlc2: GFP)* (Huang et al., 2003). Lightsheet microscopy performed at 96 hpf confirmed reduced ejection fraction in *corazoncito* embryos (Fig. 1E,F; Movies 1,2). Lightsheet microscopy afforded us sufficient resolution to visualize changes in wall thickness and cellular dimensions throughout the cardiac cycle. This technique revealed that *cora^stl325/stl325^* embryos displayed significant decreases in systolic wall thickening, longitudinal, and radial strain. Measurement of cardiomyocyte dimensions throughout the cardiac cycle indicated that *cora^stl325/stl325^* cardiomyocytes failed to shorten and thicken in systole suggestive of systolic dysfunction. Furthermore, we observed that *cora^stl325/stl325^* cardiomyocytes were shorter and thicker in diastole compared to controls indicative of concomitant diastolic dysfunction (Fig. 1F).

**Fig. 2.**
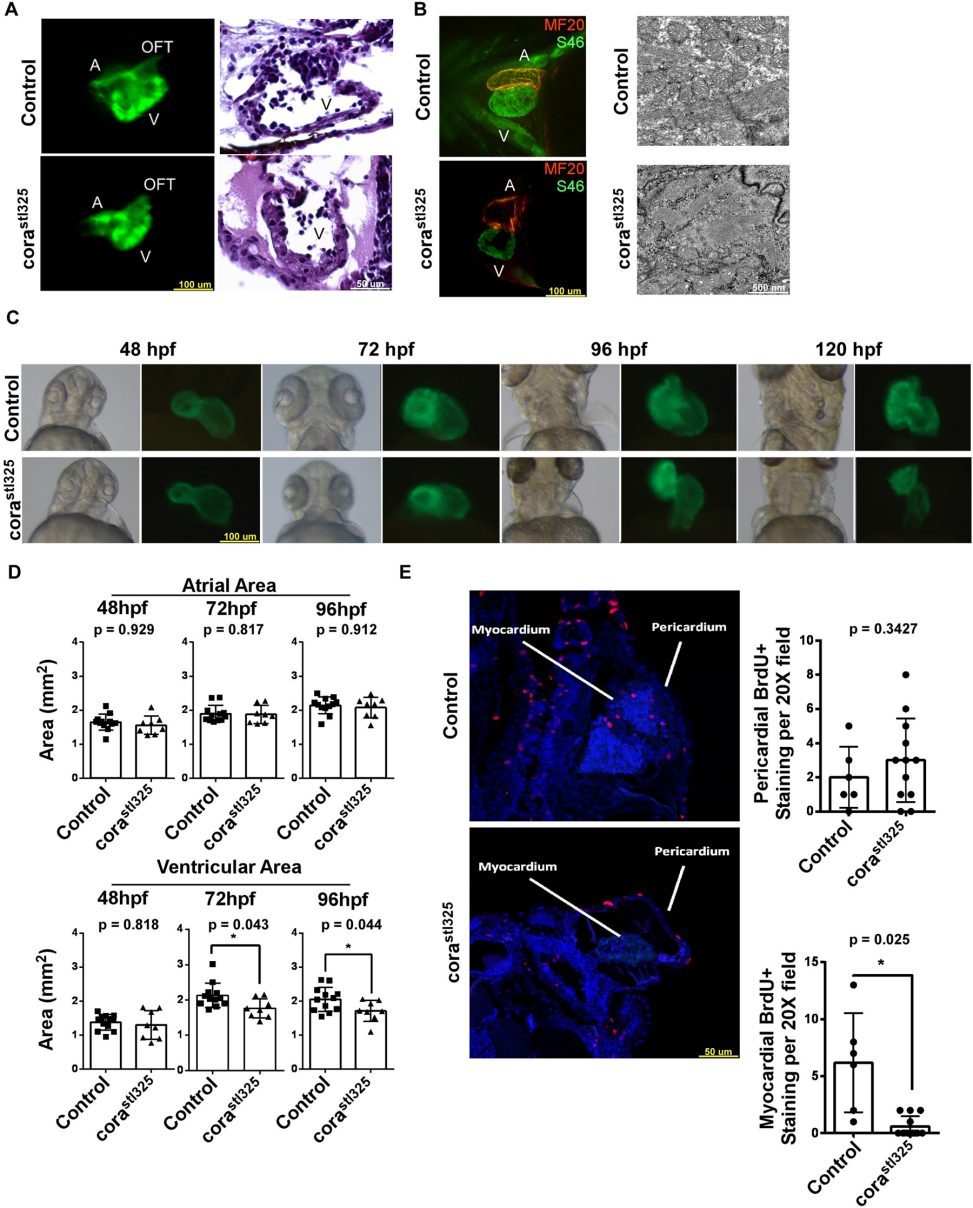
**cora*^stl325^* hearts display hypoplasia and reduced proliferation**. (A) Fluorescent microscopy images of 96 hpf embryos (left, cmlc2::GFP; *n*=8) and H&E staining (right; *n*=4). (B) Fluorescent microscopy images of 96 hpf embryos stained with the SF46 (orange) and MF20 (green) showing normal atrioventricular patterning (left). Electron microscopy showing intact sarcomeres in control and *cora^stl325^* cardiomyocytes (right; *n*=4). (C) Brightfield and fluorescence microscopy time-course of cmlc2::GFP expression between 48 and 120 hpf (Control (*n*=12) and *cora^stl325^* (*n*=8). (D) Quantification of atrial and ventricular cross-sectional areas obtained from C [Control (*n*=12) and *cora^stl325^* (*n*=8)]. (E) BrdU immunostaining (red) of 96 hpf embryos embedded in paraffin [Control (*n*=6) and *cora^stl325^* (*n*=12)].

*cora^stl325/stl325^* hearts show normal atrial and ventricular architecture, chamber patterning, and sarcomere formation (Fig. 2A,B). Serial imaging of *cora^stl325/stl325^ Tg(cmlc2:GFP)* hearts revealed diminished ventricular area compared to clutch mates beginning at 72 hpf. Atrial area did not differ between experimental groups (Fig. 2C,D). Measurement of bromodeoxyuridine (BrdU) incorporation – a synthetic nucleoside analogue used to study cell proliferation in living tissues – showed reduced abundance of replicating myocardial cells in *cora^stl325/stl325^* embryos compared to unaffected siblings. We did not observe differences in abundance of replicating pericardial cells as indicated by the number of BrdU positive cells present in the pericardium (Fig. 2E). Together, these data support the hypothesis that *cora^stl325/stl325^* embryos show signs of embryonic heart failure and ventricular hypoplasia.

### *cora^stl325/stl325^* embryos have craniofacial deformities and neuroanatomical defects

**Fig. 3.**
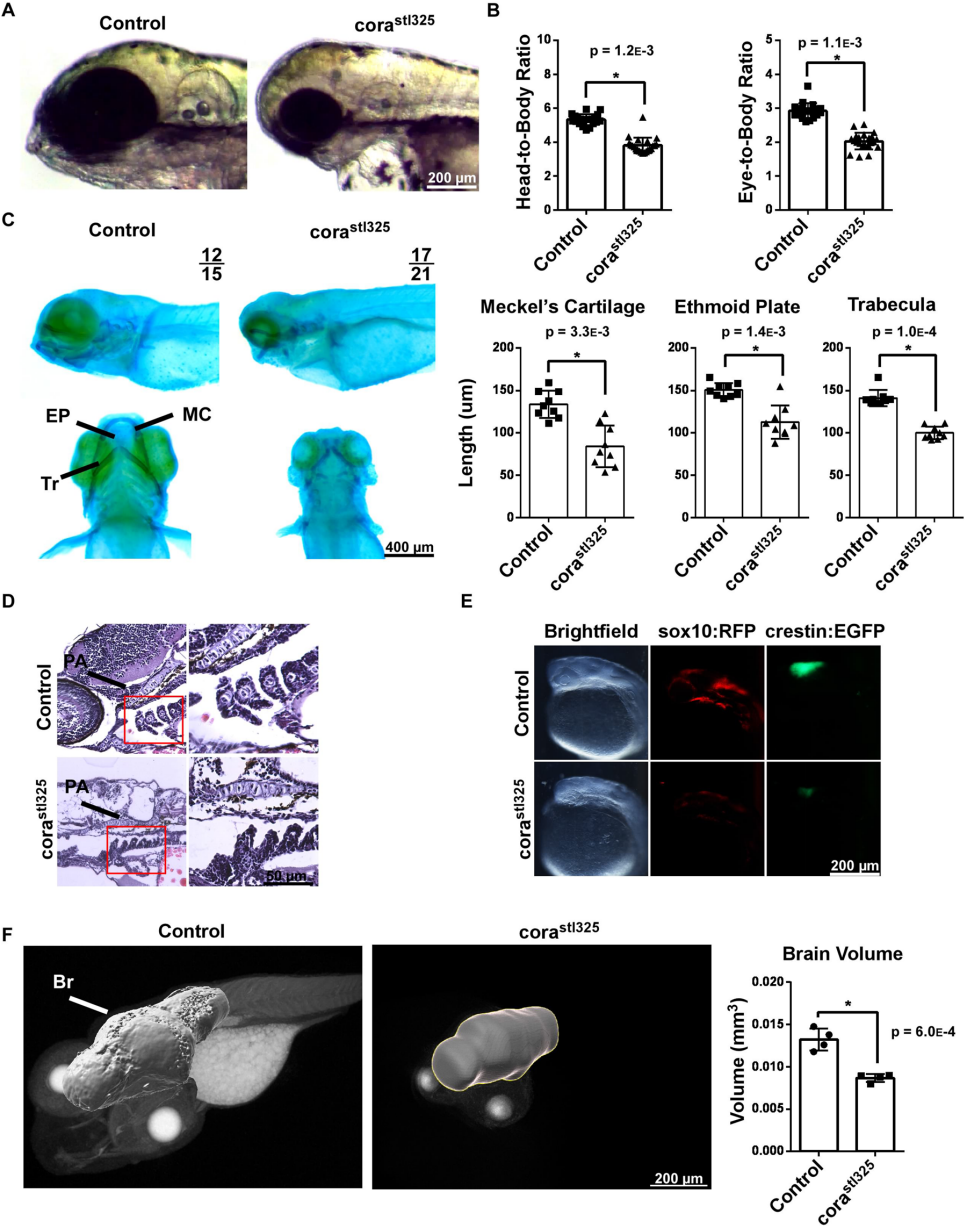
***cora^stl325^* embryos have craniofacial deformities and neuroanatomical defects**. (A) Head morphology of control and *cora^stl325^* embryos at 96 hpf. (B) Head-to-body and head-to-eye ratios. Control (*n*=13), *cora^stl325^* (*n*=9). (C) Alcian Blue staining of control and *cora^stl325^* embryos at 96 hpf (left), with quantification of major jaw elements (right). EP, Ethmoid plate; MC, Meckel's Cartilage; T, Trabeculae. *n*=9 per experimental group. (D) H&E staining of the jaw of control and *cora^stl325^* embryos at 96 hpf. (E) Fluorescence microscopy images of Tg(crestin::GFP) and Tg(sox10::RFP) control and *cora^stl325^* embryos. (F) X-ray microscopy-generated images of 96 hpf embryos (left) and quantification of tissue volume (right). Br, brain Control (*n*=4), *cora^stl325^* (*n*=4).

In addition to cardiac defects, we observed evidence of craniofacial hypoplasia in *cora^stl325/stl325^* embryos (Fig. 3A). At 96 hpf, *corazoncito* mutants displayed a 33% reduction in head-to-body and eye-to-body ratios despite slight reductions in body length seen in *cora^stl325/stl325^* embryos (Fig. 3B). We performed Alcian Blue staining to evaluate cartilaginous structures, which revealed hypoplastic jaw cartilage (Meckel's Cartilage, Ethmoid plate, Trabecula) in *cora^stl325/stl325^* embryos (Fig. 3C). Histology of 96 hpf sections suggest that chondrocytes that were specified appear morphologically similar between cora and wild-type clutchmates (Fig. 3D). Imaging of *Tg(crestin::GFP)* (migratory neural crest cell marker) and *Tg(sox10::RFP)* (cartilage progenitor marker) embryos demonstrated evidence of delayed neural crest cell migration and cartilage specification in *cora^stl325/stl325^* embryos (Fig. 3E) (Kaufman et al., 2016). X-ray microscopy further demonstrated reduced brain volume in *cora^stl325/stl325^* embryos compared to unaffected siblings (Fig. 3F). Together, these data indicate that *cora^stl325/stl325^* embryos display impaired craniofacial and neurodevelopment.

### *cora^stl325/stl325^* is a nonsense mutation in *taf5*

**Fig. 4.**
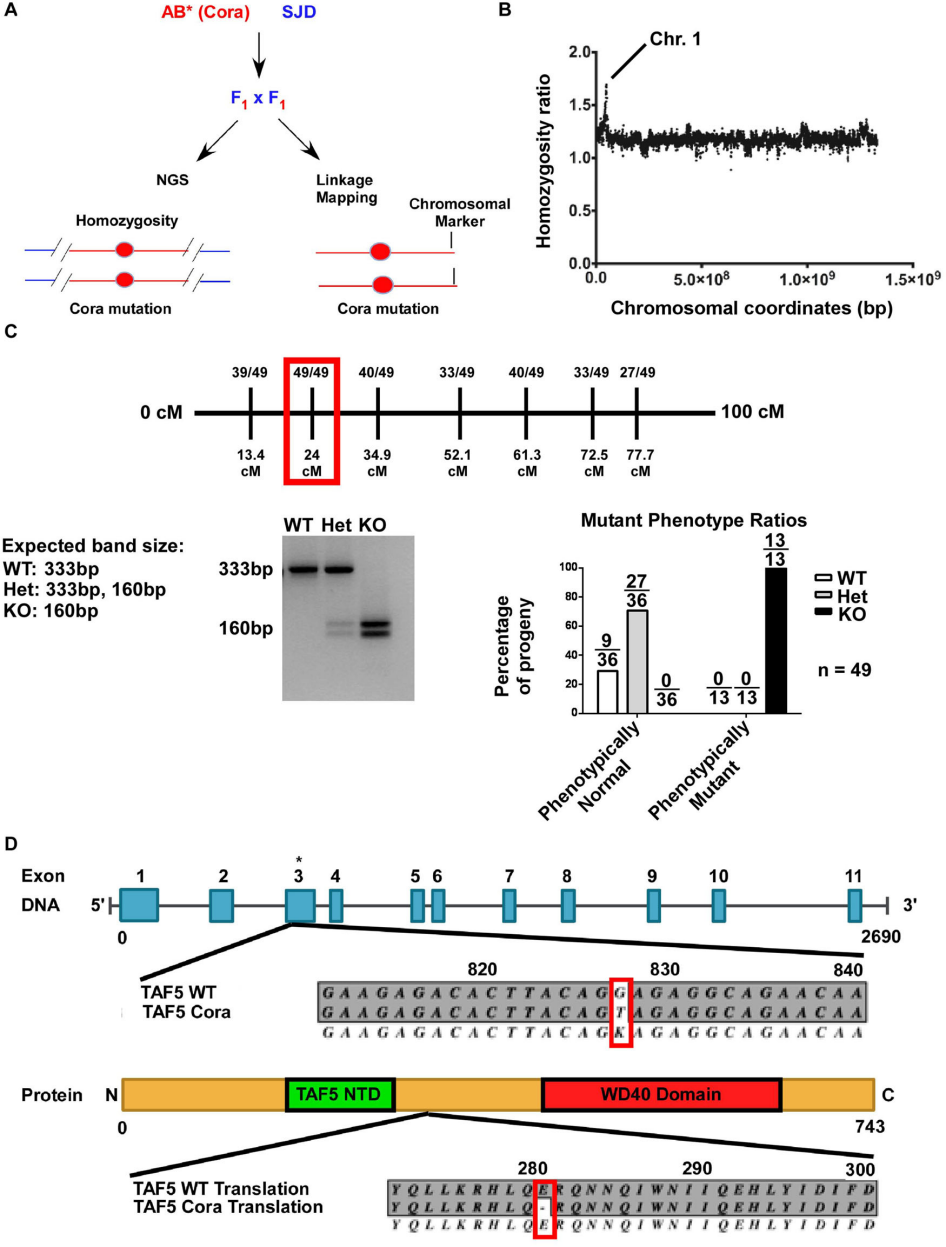
***cora^stl325^* encodes a nonsense mutation in taf5**. (A) Workflow for mapping *cora^stl325^* using whole genome next generation sequencing. (B) Manhattan plot displaying homozygosity ratio for the AB* background (y-axis) as a function of chromosome location (x-axis). A value of 2.0 indicates 100% homozygosity for the AB* background. Chr. 1: chromosome 1. (C) Fine chromosomal mapping identified a 24 cM region (red box) that was linked with the *cora^stl325^* phenotype (top). Genotyping to identify the point mutation leading to a nonsense mutation in taf5. The nonsense mutation was linked to the *cora^stl325^* phenotype (bottom). (D) Graphical representation of the taf5 genomic locus and TAF5 protein including major functional domains. Red box indicates the mutated base and resulting nonsense mutation.

To map the *cora^stl325/stl325^* mutation, we crossed *cora^stl325/stl325^* carriers (SAT background) into the SJD mapping background as described in Gray et al. (2021) (Kaufman et al., 2016). *cora^stl325/stl325^/*SJD F1 carriers were identified and self-crossed. F2 embryos were collected, pooled into *cora^stl325/stl325^* and unaffected clutch mate groups, genomic DNA isolated, and whole genome sequencing performed (Fig. 4A). We identified an area on Chromosome 1 that retained homozygosity for the AB* background that was found exclusively in embryos displaying the *cora^stl325/stl325^* phenotype (Fig. 4B). Fine chromosomal mapping identified a region on Chromosome 1 (24cM) that was closely linked with the *corazoncito* phenotype (Fig. 4C). By curating mutations found in the mapped region, we discovered a nonsense mutation in exon 3 of *taf5* (Fig. 4D). We developed a genotyping assay that specifically targeted the mutant base-pair substitution by HpyCH4III restriction digest. Genotyping of 49 embryos demonstrated 100% association between the mutated base pair and *corazoncito* phenotype, although Sanger sequencing of individual mutant embryos has not been performed (Fig. 4C).

**Fig. 5.**
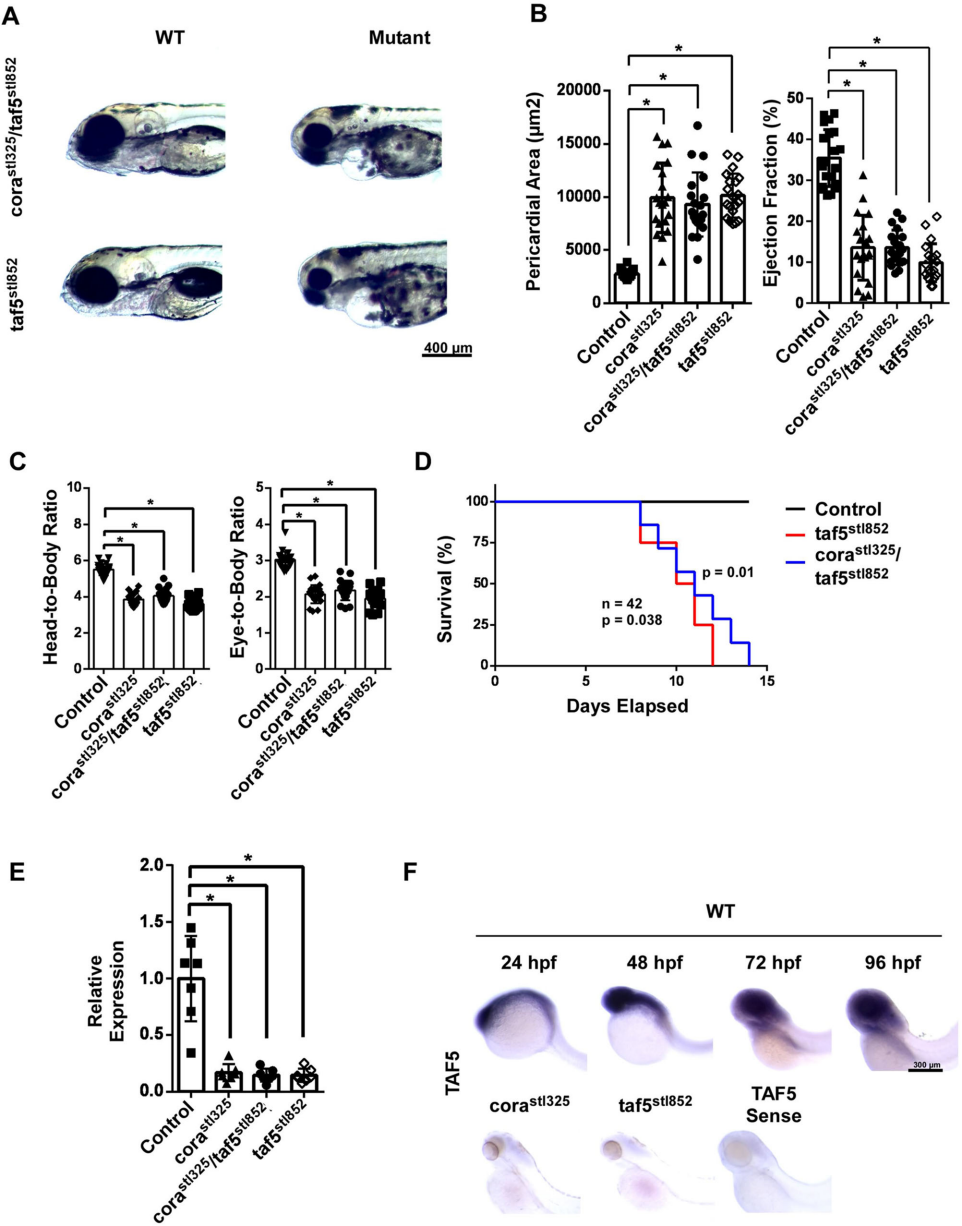
***cora^stl325^* is a null allele of taf5**. (A) Brightfield images of control, *cora^stl325^*, *taf5^stl852^*, and *cora^stl325^*/ *taf5^stl852^* embryos at 96 hpf. (B) Measurement of pericardial area and ejection fraction in control, *cora^stl325^*, *taf5^stl852^*, and *cora^stl325^*/ *taf5^stl852^* embryos at 96 hpf. *n*=22. (C) Measurement of head-to-body and head-to-eye ratios in control, *cora^stl325^*, *taf5^stl852^*, and *cora^stl325^*/ *taf5^stl852^* embryos at 96 hpf. *n*=22. (D) Survival of control, *taf5^stl852^*, and *cora^stl325^*/taf5 CRISPR embryos. *n*=42. (E) RTPCR for taf5 mRNA in control, *cora^stl325^*, *taf5^stl852^*, and *cora^stl325^*/ *taf5^stl852^* embryos at 96 hpf. *n*=7. (F) Time course of taf5 mRNA expression by whole-mount *in situ* hybridization (top) and taf5 mRNA expression in *cora^stl325^* and *taf5^stl852^* embryos at 96 hpf. Note: all brackets marked with ‘*’ represent *P*<1E-4.

To rigorously implicate *taf5* loss of function as the cause of the *corazoncito* phenotype, we generated a stable *taf5* null allele using CRISPR/Cas9-mediated mutagenesis (referred to as *taf5^stl852^*). *taf5^stl852/stl852^* embryos recapitulated the *corazoncito* phenotype of heart failure and craniofacial hypoplasia. Furthermore, *cora^stl325^* failed to complement the *taf5^stl852^* allele (Fig. 5A). Quantitative measurements of pericardial edema, ejection fraction, head-to-body, and eye-to-body ration were consistent across *cora^stl325/stl325^*, *taf5^stl852/stl852^*, and *cora^stl325^*/*taf5^stl852^* groups (Fig. 5B,C). Measurement of *taf5* mRNA expression by quantitative RT-PCR revealed marked reductions in *taf5* expression in *cora^stl325/stl325^*, *taf5^stl852/stl852^*, and *cora^stl325^*/*taf5^stl852^* embryos compared to controls (Fig. 5E). WISH further revealed that *taf5* was expressed in the whole head and whole heart region. No *taf5* mRNA was detected in *cora^stl325/stl325^* or *taf5^stl852/stl852^* embryos (Fig. 5F). Collectively, these findings support the conclusion that *taf5* loss of function is responsible for the *corazoncito* phenotype.

### *taf1* deletion recapitulates the *corazoncito* phenotype

**Fig. 6.**
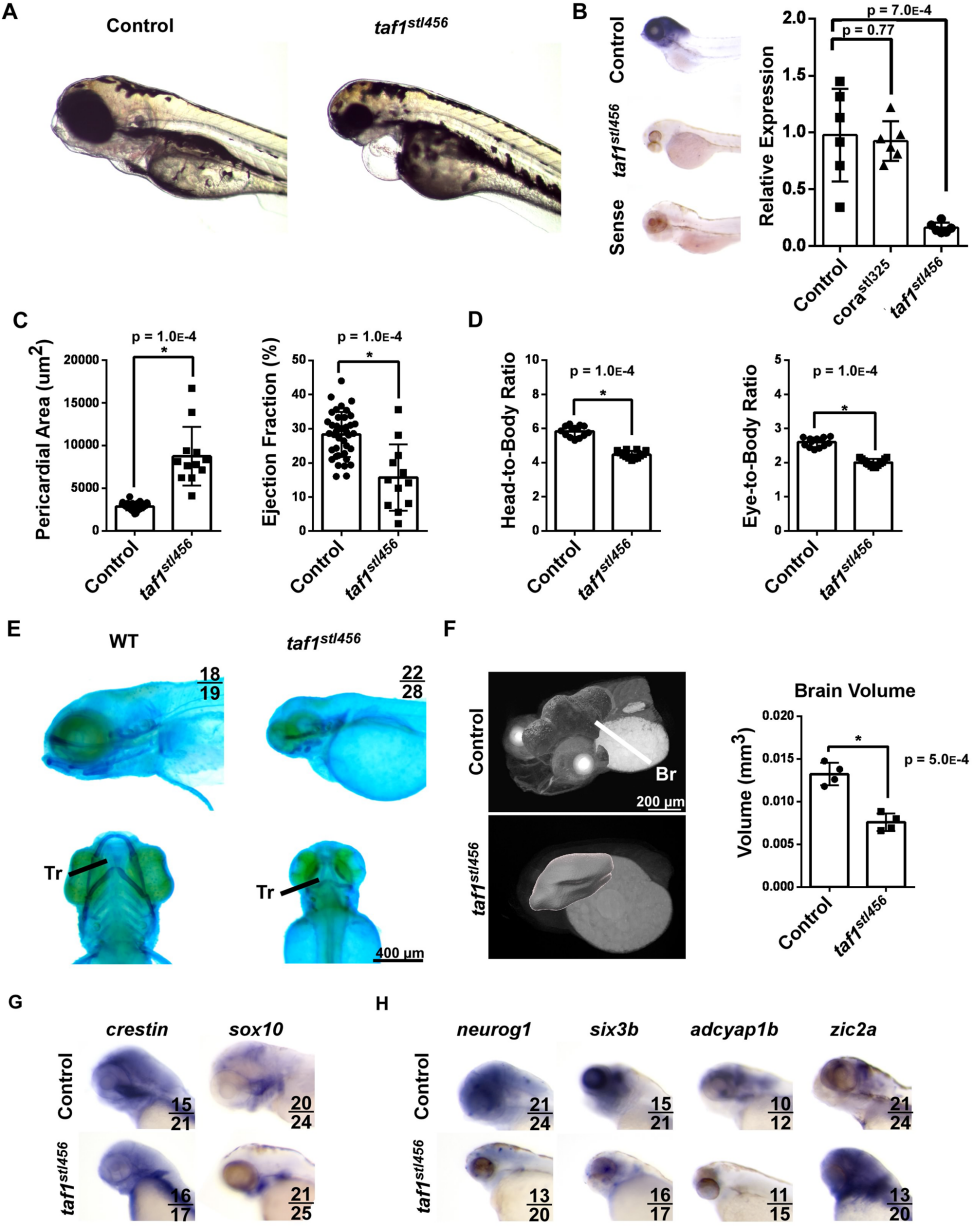
**taf1 deletion recapitulates the corazoncito phenotype**. (A) Brightfield images of control and *taf1^stl456/stl456^* embryos at 96 hpf. (B) taf1 mRNA expression measured by *in situ* hybridization (left) and RT-PCR (right). *N*=6 per experimental group. (C) Quantification of pericardial area (left) and ejection fraction (right) in control (*n*=30) and *taf1^stl456/stl456^* (*n*=12) embryos at 96 hpf. (D) Quantification of head-to-body ratio (left) and head-to-eye ratio (right) in control (*n*=21) and *taf1^stl456/stl456^* (*n*=20) embryos at 96 hpf. (E) Alcian Blue staining of control and taf1 knockout (KO) embryos. (F) X-ray microscopy-generated images of control and taf1 KO embryos at 96 hpf (left). Quantification of brain volume (right). *N*=4 per experimental group. Br, brain (red), heart (blue). (G) Whole-mount *in situ* hybridization of crestin and sox10 mRNA expression in control and taf1 KO embryos at 96 hpf. (H) Whole-mount *in situ* hybridization of neuronal (neurog1) and brain region markers (six3b, Adcyap1b, zic2a).

TAF5 acts as a scaffold protein important for the assembly of TFIID components (Bieniossek et al., 2013). Coincidentally, studies on TAF1, the catalytic subunit of TFIID, have shown that knockdown of TAF1 results in a similar phenotype of aberrant neurodevelopment (Jacobson et al., 2000). To directly compare phenotypes elicited by deletion of *taf5* and *taf1*, we utilized Cas9/CRISPR gene editing to generate a new null allele of *taf1* (referred to as *taf1^stl456/stl456^*). We observed evidence of pericardial edema, reduced ejection fraction, and craniofacial hypoplasia in *taf1^stl456/stl456^* mutant embryos relative to unaffected siblings. Consistent with these phenotypes *taf1* mRNA was expressed in the head and heart regions at 96 hpf (Fig. 6A-D). Alcian Blue staining demonstrated severe hypoplasia and loss of craniofacial cartilaginous structures (Fig. 6E), whereas X-ray microscopy showed reduced brain volume (Fig. 6F). We next performed whole mount *in situ* hybridization for markers of craniofacial development and brain patterning. WISH indicated that *crestin* expression was similar between control and *taf1^stl456/stl456^* embryos, suggesting that neural crest cells specification is not impacted by *taf1* inactivation. In contrast, *sox10* expression was markedly reduced indicating a defect in the differentiation of cartilage progenitors from neural crest cells (Fig. 6G). We also observed decreased expression of pan-neural (*neurog1*), forebrain (*six3b*), and midbrain (*adcyap1b*) markers and an expansion of the hindbrain marker, *zic2a* (Fig. 6H). Collectively, these findings indicate an overlapping phenotype between *taf1* and *taf5* mutant embryos that recapitulates key features of TAFopathies. The phenotype of *taf1* mutant embryos was comparable to that of *taf5* mutant embryos.

### *taf5 l* is dispensable for embryonic development

*taf*5 is closely related (46% amino acid identity) to an evolutionarily conserved homolog, *taf5l* (Fig. S1A). To ascertain its function and examine potential redundancy between *taf5* and taf5*l*, we generated a null allele in exon 2 of *taf5l, taf5l^stl851^*, WISH revealed that *taf5l* was expressed ubiquitously throughout WT embryos, whereas *taf5l* RNA was not detected in embryos homozygous for our nonsense allele (Fig. S1B). *taf5l^stl851/stl851^* mutant embryos were viable and did not display any evidence of craniofacial hypoplasia, pericardial edema, or reduced cardiac function at 96 hpf and grew into fertile adults. Crosses of *taf5l^stl851/stl851^* fish to test for possible maternal effects produced normal progeny, indicating that *taf5l* is dispensable for development. Furthermore, compound mutant analyses indicated that loss of *taf5l* function did not worsen craniofacial and cardiac phenotypes observed in *taf5* mutant (*corazoncito*) embryos (Fig. S1C,D) suggesting that *taf5* and *taf5l* are not redundant.

### *taf5* regulates oxidative metabolism

**Fig. 7.**
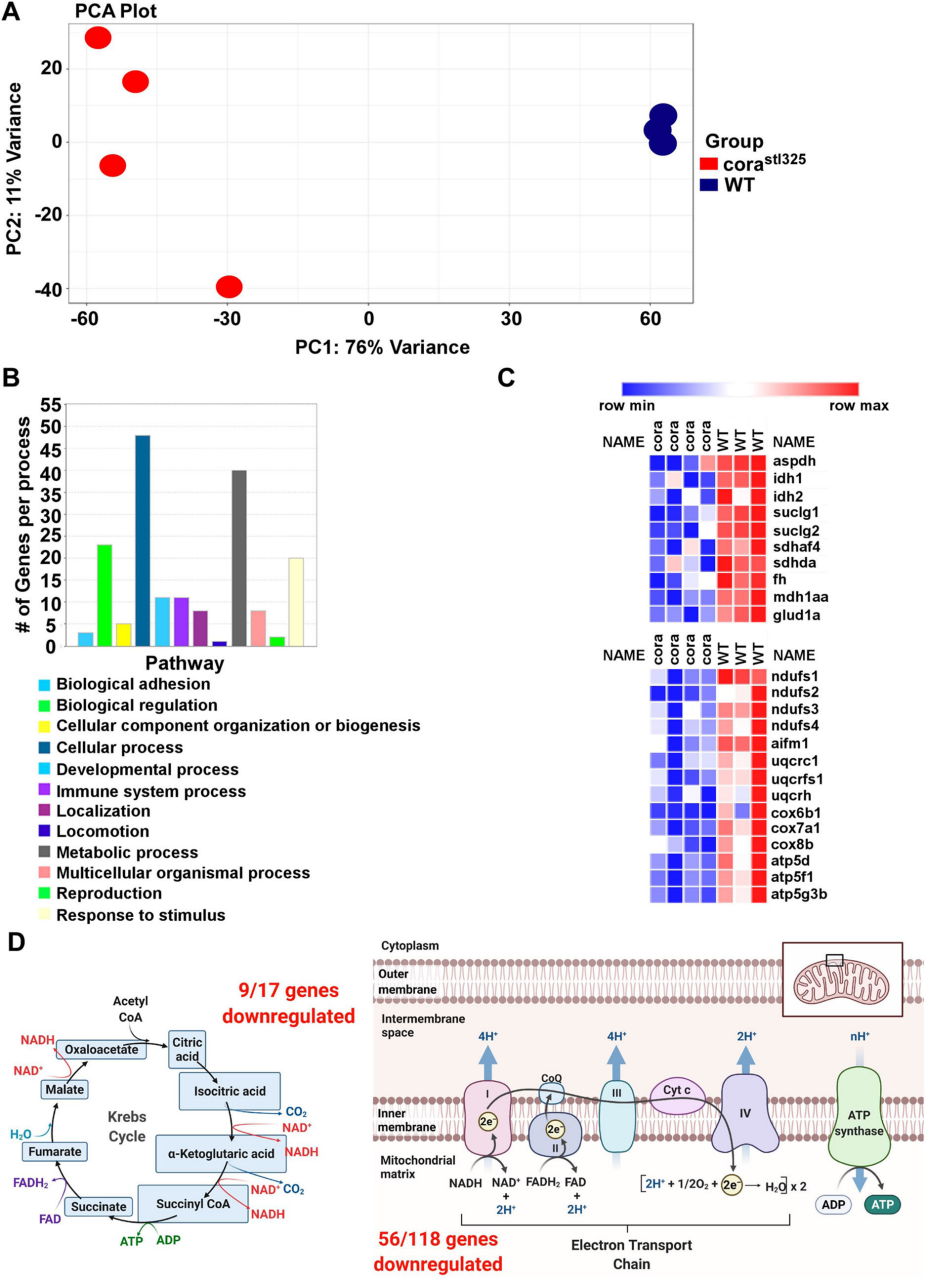
**RNA sequencing of control and *cora^stl325^* identifies derangements metabolic gene expression**. (A) Principal component analysis (PCA) plot of pooled sequencing libraries generated from control and *cora^stl325^* embryos at 96 hpf. Each dot represents a pool of 100 embryonic hearts. (B) Bar graph of the top Gene Ontology (GO) pathways associated with genes differentially expressed between control and *cora^stl325^* hearts. (C) Heat map of metabolic genes significant with significantly reduced expressed in *cora^stl325^* hearts. (D) Graphical representation of the number of differentially expressed metabolic genes associated with the TCA cycle (left) and the electron transport chain (right). Made with BioRender.

As *taf5* is a member of the TFIID complex (Patel et al., 2018) and likely regulates transcription, we performed RNAseq to investigate potential mechanisms by which *taf5* loss of function contributes to the observed cardiac phenotypes. We isolated RNA from embryonic hearts recovered from *cora^stl325/stl325^; Tg(cmlc2::gfp)* embryos and their unaffected *Tg(cmlc2::gfp)* clutchmates at 96 hpf. Control and *cora^stl325/stl325^* sequencing libraries were generated from pooled samples each containing 100 hearts. Principal component analysis (PCA) revealed markedly distinct transcriptional profiles between control and *cora^stl325/stl325^* hearts, with 2274 genes showing an log2 fold value of 1 or greater in expression (Fig. 7A). Pathway analysis suggested alterations in several distinct biological processes including metabolism (Fig. 7B). *cora^stl325/stl325^* hearts displayed downregulation of numerous genes involved in the TCA cycle (9/17 genes) and the Electron Transport Chain (56/118 genes) (Fig. 7C,D). Interestingly, other major pathways, including glycolysis (7/20), amino acid (10/24), and lipid (20/26) metabolism were significantly impacted.

**Fig. 8.**
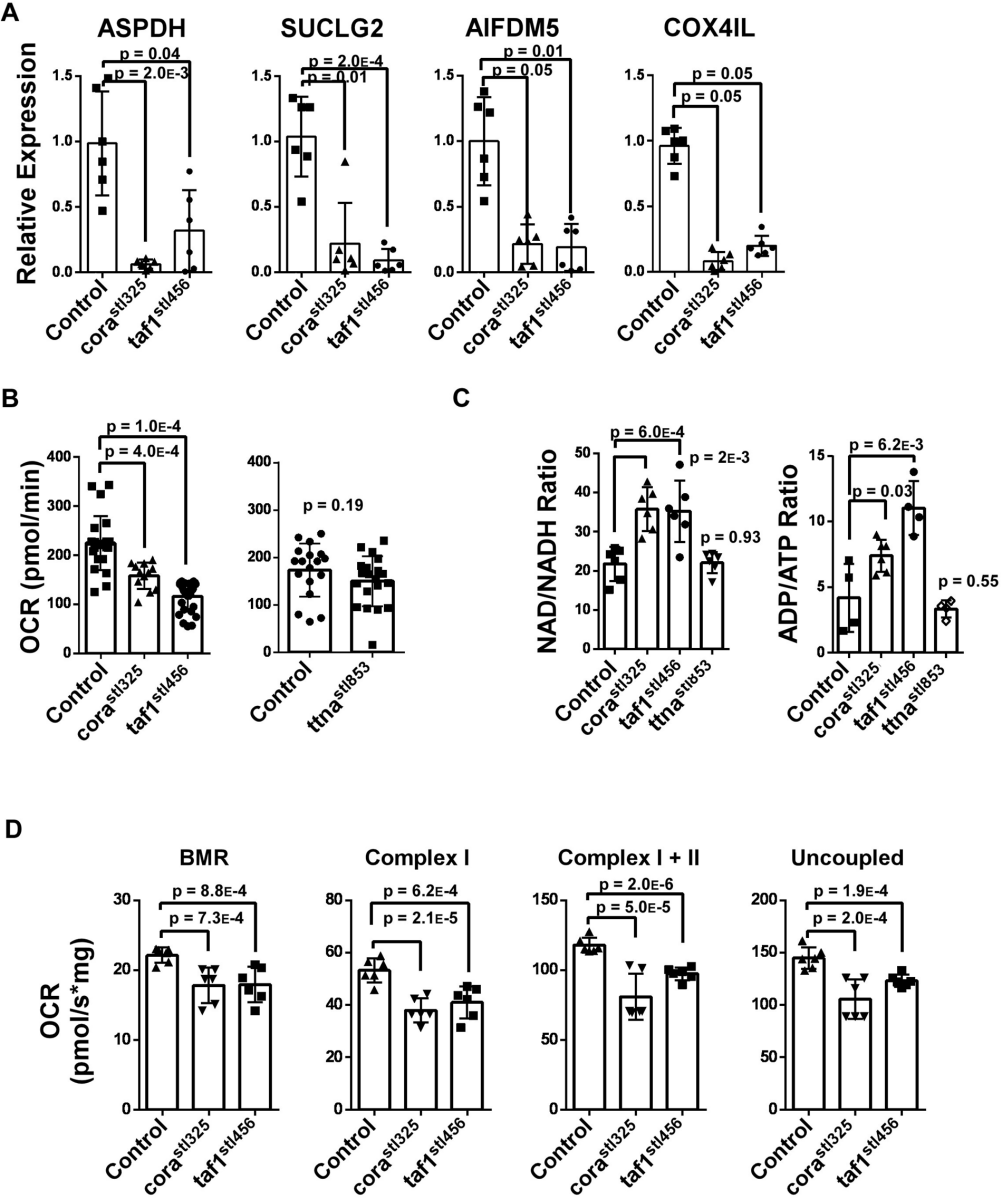
**Defective oxidative metabolism in *cora^stl325^* and taf1 knockout embryos**. (A) RTPCR showing the reduced expression of genes involved in the TCA cycle (Asdph, Suclg2) and the electron transport chain (Aifdm5, Cox4il) in *cora^stl325^* and *taf1^stl456^* embryos at 96 hpf. Each data point represents a pool of 20 embryos. (B) Reduced basal oxygen consumption rates (OCR) in *cora^stl325^* (*n*=12) and *taf1^stl456^* (*n*=12) embryos compared to controls at 96 hpf (left). Preserved basal OCR in ttn^stl853/stl853^ (*n*=20) compared to controls (*n*=18) embryos at 96 hpf (right). Data were obtained from a Seahorse XF24 Bioanalyzer. (C) Targeted metabolite assays measuring NAD/NADH (left) and ADP/ATP (right) in control, *cora^stl325^ taf1^stl456^*, and ttn^stl853/stl853^ embryos at 96 hpf. *n*=4 pools of 100 embryos per genotype. (D) Oxygen consumption rates of isolated mitochondria for fatty acid metabolism. *n*=4 pools of 100 embryos per genotype

To determine whether metabolic genes were globally downregulated, we collected RNA from control, *cora^stl325/stl325^*, and *taf1^stl456/stl456^* whole embryos at 96 hpf. Quantitative RT-PCR confirmed reduced *aspdh*, *suclg2*, *aidm5*, and *cox4il* mRNA expression in *cora^stl325/stl325^* and *taf1^stl456/stl456^* embryos relative to unaffected siblings (Fig. 8A). To discern whether altered metabolic gene expression affected metabolic function, we measured whole embryo basal metabolic rate using a Seahorse XF24 Bioanalyzer at 96 hpf. These studies showed diminished oxygen consumption rate (OCR) in *cora^stl325/stl325^* and *taf1^stl456/stl456^* embryos relative to control clutch mates. To verify that decreased basal metabolic rate was not a secondary result of heart failure, we measured basal OCR in embryos lacking *titin (ttna^stl853^)*, an established model of profound heart failure (Fig. S2) (Nechiporuk et al., 1999). Basal OCR did not differ between control and *ttna^stl853/stl853^* embryos (Fig. 8B). Reduced metabolic activity was further supported by increased NAD/NADH and ADP/ATP ratios in *cora^stl325/stl325^* and *taf1^stl456/stl456^* embryos relative to control siblings and *N2:ttna* embryos (Fig. 8C). Mitochondrial function was assessed using an Oroboros O2k-FluoRespirometer at 96 hpf. Consistent with the Seahorse data, *cora^stl325/stl325^* mitochondrial respiration showed significant perturbations at all states of respiration (Fig. 8D). Together, these results suggest that *taf1* and *taf5* are essential regulators of oxidative metabolism during embryonic development.

## DISCUSSION

While the biochemical properties of TAFs and the TFIID complex are well understood, much remains to be learned regarding their *in vivo* functions, including roles during embryonic development and tissue homeostasis as well as how pathogenic variants in TAFs contribute to diseases. Here, we leveraged several genetic zebrafish models we generated to identify a causal link between mutations in *taf1* and *taf5*, TAFopathy phenotypes, and derangements in metabolism. These studies suggest that the TAF complex does not only globally regulate transcription, but rather acts as a selective modulator of specific transcriptional programs. Furthermore, our findings provide new insights implicating defects in metabolism in the pathogenesis of TAFopathies.

TAFs are general transcription factors that are TFIID complex components which recruit RNA polymerase II to gene promoters to form the pre-initiation complex (PIC) (Patel et al., 2018). Many TAFs modulate interactions between gene-specific transcriptional activators and general transcription machinery by either stabilizing or inducing conformation chances to the PIC (Zou et al., 2015; Rhee and Pugh, 2012). Structurally, TAF5 forms a homodimer and acts as a scaffold for TFIID complex formation. Its C-terminus contains a WD40 domain, which mediates protein-protein interactions and may be important for TAF-TAF interactions (Roeder, 1996). TAF1 is the largest subunit of TFIID and contains multiple domains with enzymatic activity and chromatin interaction capabilities (Oelgeschläger et al., 1998; Wang et al., 2014). Recent studies have revealed that the TFIID complex is a three-lobed, asymmetric structure and that the TAF1 and TAF5 subunits contribute to forming this characteristic structure (Bhattacharya et al., 2007; (Oelgeschläger et al., 1998).

The alleles generated in this study for *taf5*, *taf5l*, and *taf1* all contained nonsense mutations that represent loss of function phenotypes. Interestingly, while it is believed that taf5 and taf1 operate in the same complex, deletion of one did not impact the expression of the others. Phenotypically, we show that *taf5* and *taf1* are necessary for embryonic heart, craniofacial, and brain development in zebrafish. Brightfield and fluorescent microscopy revealed classical signs of heart failure in *taf5* and *taf1* mutant (loss of function, null alleles) as measured by reduced ejection fraction, dimensional cardiomyocyte strain, and pericardial edema. Despite these functional defects, neither *taf5* nor *taf1* were necessary for heart patterning, as atrioventricular staining revealed no obvious differences in chamber specification or structural architecture. Instead, we observed decreased myocardial proliferation in *taf5* mutants. Proliferation of other structures such as the pericardium was not affected, suggesting a cell-specific role for *taf5* in myocardial proliferation. Consistent, with this finding, TAF1 upregulates cyclin D and cyclin A expression through TAF1 histone acetyltransferase activity (Jacobson et al., 2000) and TAF1 has been shown to associate with the leukemia-promoting oncogene AML1-ETO, which promotes proliferation of AML1-ETO-expressing myeloid leukemia cells. Furthermore, TAF1 is required for leukemic cell self-renewal, and its reduction promotes the differentiation and apoptosis of AML1-ETO+ myeloid leukemia cells (Gangloff et al., 2000).

We also observed significant decreases in head size, brain volume, and disruption of defined brain regions in *taf5* and *taf1* mutants. Neurodevelopmental defects have been previously observed in *taf1* mutants as evidenced by decreased head-to-body and eye-to-body ratios, and measurements of optic tectum size (Kloet et al., 2012). Loss of *TAF1* in rats alters the morphology and function of the cerebellum and cerebral cortex, and leads to hypoplasia and loss of Purkinje cells, with behavioral abnormalities paralleling that seen in TAF1/MRSX33 intellectual disability syndrome (Xu et al., 2019). TAF1 has also been linked to X-linked dystonia parkinsonism, with an alternatively spliced transcript of *TAF1* discovered in neurons (Gudmundsson et al., 2019; Grune et al., 2022; Capponi et al., 2021). These findings implicate TAF1 in early neurodevelopment and aging.

To illuminate mechanisms that contribute to TAFopathies in our zebrafish models, we performed RNA sequencing in *taf5* mutant hearts. Surprisingly, we did not detect global downregulation of transcription. Instead, we identified specific reductions in the expression of genes involved in metabolism: fatty acid oxidation, TCA cycle, and electron transport chain. Derangements in metabolism were further supported by reduced expression of key metabolic genes throughout the embryo, increased NAD/NADH and ADP/ATP ratios, and measurements of oxygen consumption at the organismal and mitochondrial levels. Importantly, these metabolic derangements were absent in *N2:TTNa* mutants with akinetic hearts indicating that metabolic impairments found in *taf1* and *taf5* embryos were not secondary to heart failure. Indeed, metabolic remodeling plays an essential role in cardiac hypertrophy and heart failure (Al Ali et al., 2021; Aneichyk et al., 2018; Ashrafian et al., 2007). Consistent with previous findings in *taf1* mutants, we observed downregulation of the cell cycle regulator *cyclin D* (*ccnd1*) in *taf*5 mutant hearts along with reduced expression of many other cyclins (*ccna1*, *ccna2*, *ccnb2*, *ccnb3*, *ccne1*). Whether heart, craniofacial, and/or brain hypoplasia is primarily caused by metabolic remodeling or a direct effect on the transcription of cell cycle regulators represents an avenue for future investigation that will require dedicated tools that dissect the gene regulatory networks controlled by TAFs and the TFIID complex.

Our study is not without limitations. We primarily focused on zebrafish models and validation of our findings pertaining to the phenotypic impact of *taf1* and *taf5* mutations and impact on metabolism in mammalian systems is an important next step. In support of our findings, previous studies have suggested that perturbations in zebrafish *taf1* result in overlapping phenotypes including reduced embryo length, underdeveloped cartilage, short pectoral fins, edema, and body axis deformations (Kloet et al., 2012). Our work adds to the understanding of the requirement for *taf1* during zebrafish development and provides new information implicating a similar requirement for *taf5* in craniofacial, neurodevelopmental, and cardiac development. The precise mechanism by which *taf1* and *taf5* specifically regulate transcriptional programs such as metabolism remains to be defined. An interesting possibility is that the TAF complex may optimize interactions between promoter and enhancer regions through the BRD complex, as TAF1 contains both TATA binding protein and BRD binding sites (Kundu et al., 2015; Doukas et al., 2007).

In conclusion, our findings establish a causal link between null mutations in *taf1* and *taf5*, phenotypes present in TAFopathy patients, and perturbations in metabolism and cell cycle-related pathways. These findings provide exciting and novel insights into how components of general transcription machinery selectively regulate specific transcriptional programs and contribute to tissue and organ maturation during embryonic development.

## MATERIALS AND METHODS

### Establishment and maintenance of zebrafish lines

*cora^stl325^* was generated through an ENU mutagenesis screen (de Bruijn et al., 2009). Transgenic reporter lines such as *Tg(cmlc2:GFP*) were obtained from other labs (Huang et al., 2003). *taf5^stl852/stl852^*, *taf5l^stl851/stl851^ taf1^stl456/stl456^* lines were generated using CRISPR/Cas9-mediated mutagenesis. Using a guide RNA targeting exon 1 of TAF5 (gRNA sequence: GGCTGCGGTGAGTGGCGATGAGG), we deleted a ∼2.5 kb region spanning exons 1 and 2. *taf5l^stl851^ was* generated using CRISPR/Cas9 (gRNA sequence: GGTGTCCGCGGCCCCGTGTCAGG) to create a 17 bp deletion that resulted in a nonsense mutation. Lastly, *taf1^stl456^* was generated by using a gRNA targeting exon 7 that created a 19 bp insertion resulting in a nonsense mutation (gRNA sequence: GGTGTCCGCGGCCCCGTGTCAGG). These larvae were grown and selected based on genotype, all of which was based on existing protocols (Li et al., 2016). Genotyping of *corazoncito* exon 3 was performed using the primers 5′-ATGCGTCATGACGTAATCACATCCAGC-3′ and 5′- TCACTGGGAGTTGTAGGAGCCTGCGGC-3′, which amplified a 333-bp region including the *corazoncito* mutation, using the restriction enzyme HpyCh4III (NEB; cat. No. R0618) that bifurcates the amplicon into ∼161-bp doublets. All lines used in this study were raised and maintained by Washington University's Fish Facility. A summary of their zebrafish husbandry guidelines can be found here (https://zebrafishfacility.wustl.edu/facility-documents/).

### Microscopy experiments

Brightfield microscopy images were taken using a Leica M80 Microscope with a mounted Leica IC80 HD camera. Fish were anesthetized in 20-30 mg/L of Tricaine in E3 water and positioned for imaging. Measurements of cartilaginous structures, head and eye components, ejection fraction and pericardium were performed with ImageJ (Collins, 2007). Ejection fraction was measured by measuring the area of the ventricle at systole and diastole. We used these area measurements to find the difference in area, which was then divided by the ventricle area during diastole.

Fluorescence microscopy was performed using a Zeiss Lightsheet 7 in the Washington University Center for Cellular imaging (WUCCI). Sample preparation protocol can be found here (https://www.iob.uu.se/digitalAssets/576/c_576400-l_1-k_protocol-for-lightsheet-z.1-using-zebrafish.pdf). For Zeiss lightsheet microscopy, larvae were anesthetized in 20-30 mg/L of Tricaine in E3 water, and then placed in a 1% low-melting agarose solution. This larvae/agarose combination was then inserted into a black glass capillary, after which larvae hearts were imaged.

X-ray microscopy was performed using a Zeiss Xradia Versa 520 XRM microscope at WUCCI (Bayguinov et al., 2020). Zebrafish larvae were fixed in 4% paraformaldehyde, then transferred to Lugol's Iodine for 3 weeks, after which samples were imaged. All imaging analysis was performed using Imaris.

### Whole-mount staining

Zebrafish larvae were fixed overnight at 4°C using 4% paraformaldehyde, then washed in PBS+0.1% Tween. These embryos were then placed in 100% methanol and stored at −20°C. Alcian Blue staining was performed as previously described (Walker and Kimmel, 2007). Zebrafish larvae were imaged and staged in 100% glycerol+KOH using a Zeiss Discovery V.12 stereomicroscope.

Whole-mount *in situ* hybridization was performed according to established protocols (Cunningham and Monk, 2018). Briefly, probes were generated through PCR amplification of the coding sequences of the genes of interest using AB* cDNA libraries, followed by standard TOPO cloning (Invitrogen; cat. No. K4500) (Table S1). Transformation was performed using TOP10 chemocompetent cells (Thermo Fisher Scientific; cat. No. C505003), with confirmation of probe generation and correct orientation performed through Sanger sequencing. Once probes were validated, fixed embryos were rehydrated in a methanol/PBS gradient, permeabilized at room temperature (RT) with 10 μg/ml proteinase K, and left to fix in 4% paraformaldehyde, after which they were transferred to hybridization buffer (65% formamide, 5× SSC, 0.1% Tween, 50 μg ml^−1^ of heparin, 500 μg ml^−1^ of RNase-free tRNA adjusted to pH 6.0 by adding citric acid) containing 3 ng/μl probe overnight at 70°C. These embryos were then washed with hybridization buffer/PBS, followed by PBS washes, then incubated at 4°C overnight with 1:5000 anti-digoxigenin antibody (Roche, Catalog # 11093274910). Embryos were then washed with PBS and stained using BM Purple until expression could be clearly observed (Roche, Catalog # 11442074001).

### Next generation sequencing and RNA sequencing

Genomic DNA and cDNA obtained from *Corazoncito* and their unaffected clutchmates was submitted to the Genome Technology Access Center for whole genome sequencing and RNA sequencing. For whole genome sequencing and mutation mapping, we used an existing pipeline as described here (Sanchez et al., 2017). Briefly, 1 µg larvae gDNA was extracted and submitted to the Genome Technology Access Center (GTAC) at Washington University for whole genome sequencing where they were bar-coded and pooled, and paired-end sequencing was performed in a single lane of a HiSeq2500 or 3000 (Illumina). Reads were aligned using NovoAlign (Novocraft), and variants were called using SAMtools. Our pipeline involved using three scripts ‘ChromSplit’, ‘Allele Ratio Calculator’ (ARC), and ‘SNPFilter’ to split variants into chromosome-specific data files, calculate mutant allele frequency at each point of variation between groups, and omit SNPs that have been previously annotated. Manhattan plots were generated using GraphPad Prism). Chromosomal fine mapping was performed using established markers for chromosome 1 (Table S2). RNA sequencing pathway analysis was performed using the Shiny Transcriptome Analysis Resource Tool (Nelson et al., 2017). Pathway analysis was performed using Gene Ontology Resource (http://geneontology.org/). Heat maps were generated using Phantasus (https://artyomovlab.wustl.edu/phantasus/). FASTQ files are available (GEO GSE230183).

### RT-PCR

Primers were generated through Integrated DNA Technologies' PrimerQuest tool (https://www.idtdna.com/pages/tools/primerquest) (Table S3). RT-PCR was performed with standard conditions. RNA was extracted from zebrafish larval tissue using the RNeasy RNA mini kit and Tissue Lyser II (Qiagen). RNA concentration was measured using a nanodrop spectrophotometer (Thermo Fisher Scientific). cDNA synthesis was performed using the High Capacity RNA to cDNA synthesis kit (Applied Biosystems). cDNA was synthesized using the iScript^™^ Reverse Transcription Supermix (Bio-Rad) and pre-amplified using the Sso Advanced PreAmp Supermix kit (Bio-Rad). Quantitative real time PCR reactions were prepared with sequence-specific primers (IDT) with PowerUP^™^ Syber Green Master mix (Thermo Fisher Scientific) in a 20 μl volume. Real time PCR was performed using QuantStudio 3 (Thermo Fisher Scientific). mRNA expression was normalized to HPRT. All RT-PCR assays were performed with appropriate quality controls including melt curves and negative controls.

### Metabolism assays

Zebrafish larvae basal metabolic rate was measured in a Seahorse XF24e extracellular flux analyzer through modification of previously established protocols (Stackley et al., 2011). One 96 hpf embryo per well was used to measure the oxygen consumption rate at physiologically relevant temperature (28.5°C), with all other conditions held constant.

Targeted metabolite assays for NAD/NADH (Biovision; cat. No. K337) and ADP/ATP (Millipore-Sigma; cat. No. MAK081-1KT) were used. ATP extraction from tissues using PCA was performed according to the protocol supplied by the manufacturer. Larval tissue pools of 20 mg were flash frozen and homogenized with 200 μl of ice-cold homogenization buffer (0.25 mol L^−1^ sucrose and 10 mmol L^−1^ HEPES–NaOH, pH 7.4) by Tissue Lyser II (Qiagen) using one cycle of 30-s homogenization and 30-s cooling. After homogenization, the homogenate was centrifuged at 1000×***g*** for 10 min at 4°C. One hundred microliters of the supernatant was quickly added to an equal volume of ice-cold 10% PCA and shaken for 20-s. The supernatant was then transferred into a 2.0-mL microtube for centrifugation (10,000×***g*** for 10 min at 4°C), and 50 μl of supernatant was collected and added to 50 μl of 1 mol L^−1^ Tris–acetate buffer (pH 7.75) for neutralization. Ten microliters of aliquot from the supernatant was used in 96-well plates for luciferin–luciferase assay, which was performed using a TECAN m200 Infinite Pro plate reader.

NAD/NADH assay samples were prepared according to the manufacturer’s protocol. Twenty milligram samples of larval tissue were washed with ice cold PBS, after which 400 μl of NAD/NADH Extraction Buffer were added to the samples that were homogenized by Tissue Lyser II (Qiagen) using one cycle of 30-s homogenization and 30-s cooling. After homogenization, the homogenate was centrifuged at 24,000×***g*** for 10 min at 4°C. Supernatant was transferred to a new tube, with 200 μl of this extract placed into a separate tube that was heated to 60°C for 30 minutes in a water bath. Both the non-decomposed and decomposed samples were added to a 96-well plate to be processed with kit reagents and OD_450_ read by a TECAN m200 Infinite Pro plate reader.

### Isolation of mitochondria from zebrafish embryos

For mitochondrial isolation, anesthetized pools of 100 96 hpf embryos were collected and washed in mitochondrial isolation buffer (MIB 0.21 M mannitol, 70 mM sucrose, 0.1 mM potassium-EDTA, 1 mM EGTA, 10 mM Tris-HCL, pH 7.4) for each genotype and were homogenized at 70 rpm for 20 strokes on ice in a 4 ml glass homogenizer at a ratio of 10 mg tissue per mL of MIB. The homogenate was transferred to 1.7 ml microcentrifuge tubes and centrifuged for 10 min at 600×***g***. Subsequently, the supernatant was centrifuged for 10 min at 7200×***g*** to pellet mitochondria. The pellet was then resuspended and washed twice in MIB, with the final pellet reconstituted in 500 μl of MIB.

### Mitochondrial respiration assays for assessment of metabolic function

Protein concentration was determined using the BCA method per the manufacturer's instructions (Thermo Fisher Scientific). Mitochondria (300 ng protein) were placed in 2 ml mitochondria respiration buffer (MIR05: 0.5 mmol/L EGTA, 3 mmol/L MgCl_2_, 60 mmol/L K-lactobionate, 20 mmol/L taurine, 10 mmol/L KH_2_PO_4_, 20 mmol/L HEPES, 110 mmol/L sucrose, and 1 g/L BSA [pH 7.1]) and loaded into an Oxygraph-2k respirometer (Oxygraph-2k with O2k-Fluorescence module; Oroboros Instruments, Innsbruck, Austria) at 28.5°C with continuous stirring for the measurement of O_2_ flux. First, malate (1 mM) was added for depletion of endogenous substrates. Octanoylcarnitine (30 μM) was then added as a fatty acid substrate. Adenosine disphosphate (ADP; 5 mM) was added to induce state 3 respiration, with glutamate (10 mM) and succinate (10 mM) subsequently added for electron input in complex I and complex II, respectively. Cytochrome C (10 μM) was added to assess outer mitochondrial membrane integrity, and maximal oxygen flux rates were measured using chemical uncoupler carbonylcyanide-4-(trifluoromethoxy)-phenylhydrazone (FCCP), which was titrated (0.25 μM per addition) until no further stimulation of respiration could be detected.

### Statistics

One-way ANOVA tests were used for ejection fraction, edema, qPCR, and metabolic experiments. Log-rank test was used for survival curves. RNA-Seq data was analyzed through START (https://nasqar.abudhabi.nyu.edu/STARTapp/) and Gene Ontology (http://geneontology.org/).

## Supplementary Material

10.1242/biolopen.059905_sup1 Supplementary information
