# Supplementary material for: Deletion of taf1 and taf5 in zebrafish capitulate cardiac and craniofacial abnormalities associated with TAFopathies through perturbations in metabolism
Source: Biol Open. 2023 Jul 13;12(7):bio059905. doi: 10.1242/bio.059905 (PMC10354717; doi:10.1242/bio.059905)
Supplement: Supplementary information [file biolopen-12-059905-s1.pdf]

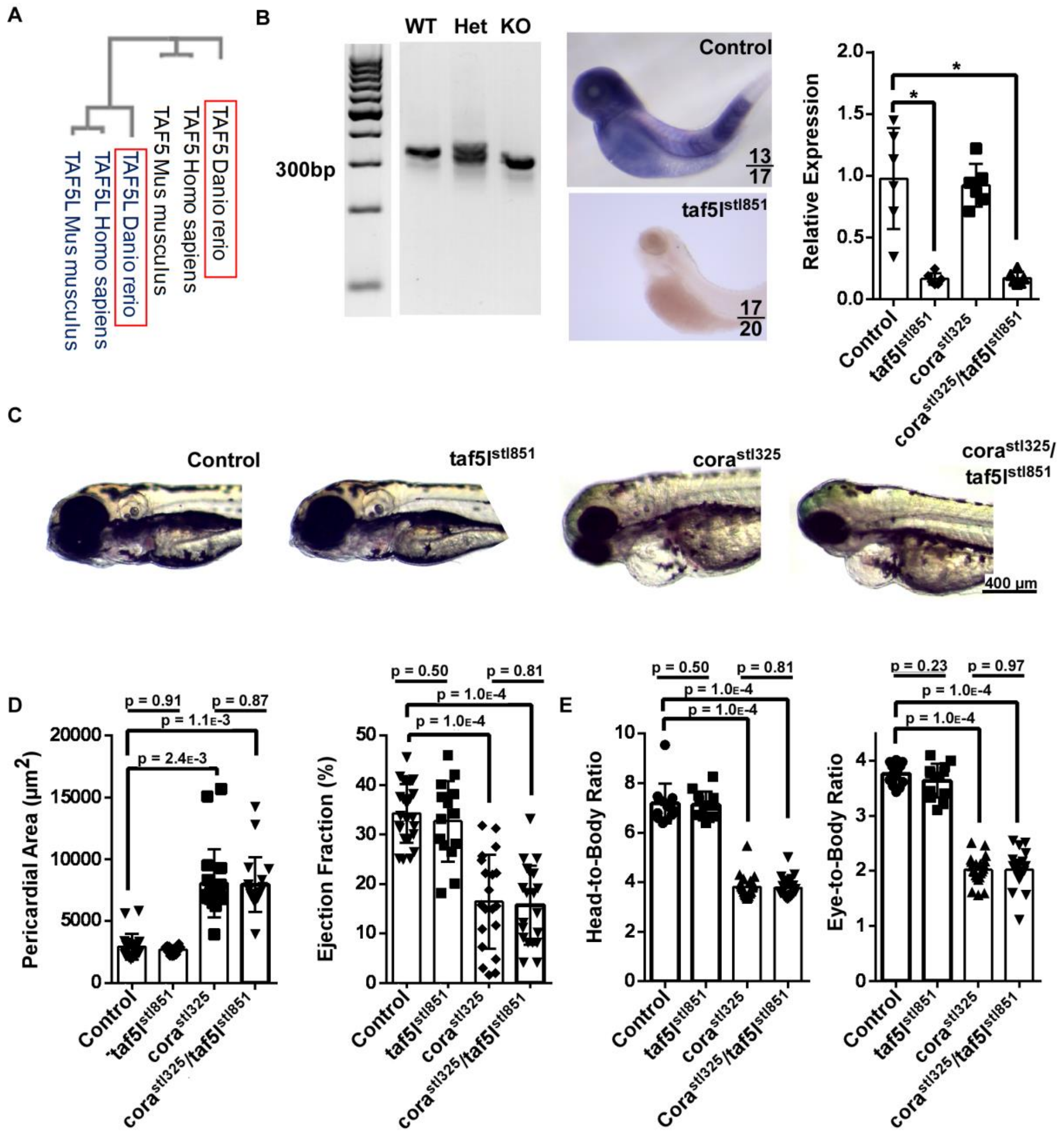

Fig. S1. Assessment of redundancy between *taf5* and *taf5l*. **A)** Dendrogram showing evolutionary conservation between TAF5 and TAFL between human, mouse, and zebrafish. **B)** Generation of *taf5l*$^{\mathrm{stl851}}$ zebrafish. Genotyping strategy to identify control, heterozygous, and homozygous *taf5l*$^{\mathrm{stl851}}$ embryos (left). In situ hybridization showing the absence of *taf5l* mRNA in *taf5l*$^{\mathrm{stl851}}$ embryos at 96 hpf. RT-PCR data showing absence of TAF5L expression in *taf5l*$^{\mathrm{stl851}}$ (right). Note: brackets marked with "*****" represent p < 1E-4 **C)** Brightfield images of control, *taf5l*$^{\mathrm{stl851}}$, *cora*^{stl325}, and *cora*^{stl325}/ *taf5l*$^{\mathrm{stl851}}$ embryos at 96 hpf. **D**) Measurement of pericardial area, ejection fraction, head-to-body, and eye-to-body ratios in of control, *taf5l*$^{\mathrm{stl851}}$, *cora*^{stl325}, and *cora*^{stl325}/ *taf5l*$^{\mathrm{stl851}}$ embryos at 96 hpf. Each data point represents an individual embryo.

Biology Open • Supplementary information

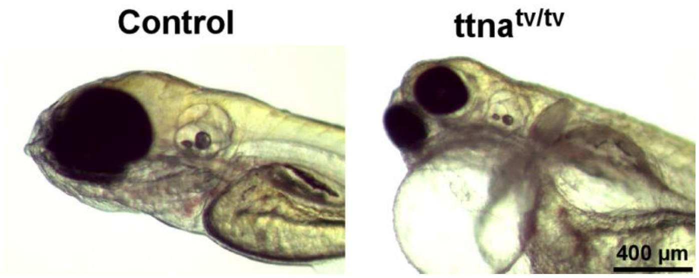

Fig. S2. Brightfield images of ttna$^{tv/tv}$ and control clutchmates showing profound signs of heart failure including pericardial and body wall edema Scale bar = 400 um.

Table S1. RT-PCR Primers

Gene	Primer Name	Sequence
**Taf5**	Taf5 F	AAAGCCTTGGCTTCGCTCTGC
	Taf5 R	CCAGCTCCAGGTACATGTGGAC
**Taf5l**	Taf5l F	AAAACCAGTTCTCCAGGCTAC
	Taf5l R	GAGGAAGAGCGGGAACAAG
**Taf1**	Taf1 F	GGTGTCTGGTGAAGGTGATAAA
	Taf1 R	GGGACACCAAGCATGTCATA
**Aspdh**	Aspdh F	CTACCACGTTGTGGAAGTAGAC
	Aspdh R	GCTGCTCCAGAATGAATTGTATG
**Suclg2**	Suclg2 F	GACAGGCTGCAGATCAGATTA
	Suclg2 R	ACTTGTCCCTCAGGTGTTTC
**Aifdm5**	Aifdm5 F	CATGGAAGTGGCAGCTTATTTG
	Aifdm5 R	CGTCATGGTGACCTTTCCTATC
**Cox4il**	Cox4il F	CACGCTTTAACAGACCCATAGA
	Cox4il R	CACGAGAAGACAGCATCTTGA

Biology Open • Supplementary information

Table S2. Primers to generate whole mount in situ hybridization Probes

Gene	Primer Name	Primer Sequence
Taf5	Taf5 F	CACCTGGCCTACTGGATGACGTAATCACATCC
	Taf5 R	TCAGATGCATCATGTTTTAAAGAGCAA
Taf5l	Taf5l F	CACCAAGAAGAAAACAGCGCGACCAATGG
	Taf5l R	CCTGCTCACTGCTCCTGCTTCTCCATGG
Taf1	Taf1 F	GCTCAGTGAGTCCAGGCAGG
	Taf1 R	GTGAGTCCAGGCAGGGC
Crestin	Crestin F	AGCCTACTACAGCAGACTCA
	Crestin R	GAGGTTTCTCAGGAAGAGGATTT
Sox10	Sox10 F	CACACATGTCTCCACCCAATTA
	Sox10 R	GCGCTCACATTTACTGCTTTC
Neurog1	Neurog1 F	CGTCGTCTTCATCACCGTCTTACTGC
	Neurog1 R	GGATCAGTCGGACAGATGAGGGTTTCTTCG
Six3b	Six3b F	CAGGTCTCCTTTAGAGCTTTATCC
	Six3b R	CGTTCCTTGAAACAGTGCGTCTTCTG
Adcyap1b	Adcyap1b F	CGAGTGCTGCTGATTTCATCG
	Adcyap1b R	GATAACTGAACCAGGATGTCCC
Zic2a	Zic2a F	GGACAGAGACTTGAGTTTAGCGC
	Zic2a R	GCTGCCTCATGTAGCGAAAGAAGGC

Table S3. zMarker Primers

Primer Name	Primer Seq
z9217F	GCCCAGCCCTCTGTACATTA
z9217R	AGGAGAATCGCCATTTCTTG
fc27h08F	TCTGTTTTTGCTTTTTCTTTCTTTC
fc27h08R	CGCCCCGAACTACACAGTAAC
z1154F	TCATGATTGTTTGGAATGTAATAGTG
z1154R	TTGAGCGGTAGTCTTCTACGC
z11154F	GATCGGGATTTCAAAGCAGA
z11154R	GAGCGAAACGAAAGAAGTGC
z6802F	TCCAAGCACTTCTCCATTCC
z6802R	CTCCGACGCGATCATATTAA
z6283F	TCAACACGCTTTATAGGGGG
z6283R	TTGTGACGCCTCTGACTGTC
z22347F	GAGCAGCAGAGAGCAGGAGT
z22347R	TTGGTGTTTGCTGTCCTCTG

Biology Open • Supplementary information
